# Translation of Sindbis Subgenomic mRNA is Independent of eIF2, eIF2A and eIF2D

**DOI:** 10.1038/srep43876

**Published:** 2017-02-27

**Authors:** Miguel Angel Sanz, Esther González Almela, Luis Carrasco

**Affiliations:** 1 Centro de Biología Molecular Severo Ochoa (CSIC-UAM) Universidad Autónoma de Madrid, 28049, Madrid, Spain

## Abstract

Translation of Sindbis virus subgenomic mRNA (sgmRNA) can occur after inactivation of eIF2 by phosphorylation in mammalian cells. Several studies have suggested that eIF2 can be replaced by eIF2A or eIF2D. HAP1 human cell lines knocked-out for eIF2A, eIF2D or both by CRISPR/Cas9 genome engineering were compared with wild-type (WT) cells to test the potential role of eIF2A and eIF2D in translation. Sindbis virus infection was comparable between the four cell lines. Moreover, synthesis of viral proteins during late stage infection was similar in all four cell lines despite the fact that eIF2α became phosphorylated. These findings demonstrate that eIF2A and eIF2D are not required for the translation of sgmRNA when eIF2α is phosphorylated. Moreover, silencing of eIF2A or eIF2D by transfection of the corresponding siRNAs in HAP1 WT, HAP1-eIF2A^−^ and HAP1-eIF2D^−^ cells had little effect on the synthesis of viral proteins late in infection. Modification of AUG_i_ to other codons in sgmRNA failed to abrogate translation. Sindbis virus replicons containing these sgmRNA variants could still direct the synthesis of viral proteins. No significant differences were found between the cell lines assayed, suggesting that neither eIF2A nor eIF2D are involved in the translation of this sgmRNA bearing non-AUG codons.

Upon infection of susceptible cells, animal viruses express their genomes to synthesize a number of viral proteins involved in genome replication and in the modulation of many cellular functions. Viral proteins are produced by translation of mRNAs that have evolved several structural characteristics to compete with cellular mRNAs. Accordingly, translation of some viral mRNAs follows a variety of virus-dependent non-canonical mechanisms. Sindbis virus (SINV), an alphavirus, has two different mRNAs that are translated at different times during infection. SINV genomic RNA is of positive polarity and is immediately translated early during infection to produce non-structural proteins (nsP1–4) that participate in genome replication and transcription^1^,^2^. The recognition of an internal promoter in the negative strand RNA that is complementary to the genomic RNA is necessary to initiate synthesis of subgenomic mRNA (sgmRNA), the most abundant viral mRNA during the late phase of infection that directs the synthesis of structural proteins when cellular translation is drastically inhibited. SINV sgmRNA (4,105 nt without the poly(A) tail) devotes the bulk of its sequence (3,738 nt) to encode the structural proteins C-E3-E2-6K-E1, initially synthesized as a polyprotein. The coding sequence is flanked by two untranslated regions (UTR). The 5′-UTR (49 nt) represents the leader sequence and contains a cap structure at its 5′ end. This leader sequence confers eukaryotic initiation factor complex, eIF4F, independence and is implicated in the shut-off of host translation^3^,^4^. It has been suggested that 80S ribosomes could directly interact with the AUG initiation codon without scanning by the preinitiation complex^5^; however, it has been demonstrated that scanning of the leader sequence is obligatory for sgmRNA translation^6^. For this scanning to occur, recognition of the cap-structure by eIF4E is likely not necessary since cleavage of eIF4G by poliovirus 2A^pro^ or human immunodeficiency virus protease does not impede sgmRNA translation in SINV-infected cells^3^,^7^. The 3′-UTR (323 nt) can be divided into three different domains. One region of 19 nt near to the poly-(A) tail is involved in RNA replication^8^,^9^, while an A/U-rich domain of about 60 nt interacts with the host protein HuR, participating in mRNA stability^10^,^11^,^12^. The 240-nt-region located between the end of the coding region and the A/U-rich domain contains three repeated sequences^13^ and is involved in the stimulation of translation in insect cells^14^. This structure at the 3′-UTR therefore constitutes a translational enhancer that functions in a cell-specific manner. Besides the aforementioned structures present at the 5′-and 3′-UTR, a hairpin in the coding sequence can be found located 77–139 nt from the 5′ end^15^. This downstream hairpin (DLP) is not a true enhancer of protein synthesis, but instead is involved in conferring eIF2-independent translation of sgmRNA in infected mammalian cells^16^,^17^,^18^. A second important function of the DLP is to signal the precise codon at which to start translation^7^. Thus, DLP disorganization does not diminish translation in PKR-deficient mouse embryonic fibroblasts, but its translation is obstructed when eIF2 is phosphorylated^17^,^18^. It is therefore interesting to note that sgmRNA translation can take place without an intact eIF4F complex and after eIF2 inactivation by eIF2α phosphorylation in SINV-infected cells, despite the fact that this mRNA does not contain an IRES motif^19^ and is translated by a scanning mechanism^6^.

The possibility that eIF2 function is replaced by other cellular factors has been proposed^5^,^17^. One such possibility is that eIF2A substitutes for eIF2 in SINV-infected cells. eIF2A is a 65 kDa protein that was described several years ago, but its precise function in mammalian cells remains unclear and deletion of the yeast orthologue has no effect on cell viability, although sporulation is affected^20^. Early results demonstrated that eIF2A can interact with Met-tRNA_i_^Met^ to bind it to the ribosome^21^; however, this binding was much less efficient than that observed using genuine eIF2 on artificial templates and eIF2A was unable to promote the binding of Met-tRNA_i_^Met^ to globin mRNA^22^. More recent results from mammalian cells suggest that eIF2A is involved in the translation of some specialized cellular mRNAs that initiate translation with non-AUG codons^23^,^24^. The finding that yeast eIF2A is found in 40S and 80S ribosomes suggests its involvement in the initiation of at least some mRNAs^25^. Indeed, eIF2A represses the translation of several yeast mRNAs bearing IRES structures^26^,^27^. Accordingly, the functioning of eIF2A in yeast and mammalian cells may differ.

A second possibility is that eIF2D initiates sgmRNA translation in place of eIF2^5^. eIF2D has two functional domains: PUA and SUI1^28^. The PUA domain is an RNA-binding domain found in several enzyme families, such as those that modify tRNA. The SUI1 domain, which is also found in eIF1, is involved in the recognition of the translation initiation codon. eIF2D is an initiation factor that was erroneously named ligatin^5^, but further studies demonstrated that eIF2D and ligatin were two different proteins^29^. Initially, eIF2D was purified from rabbit reticulocyte lysates as a 65 kDa protein that could displace deacylated tRNA and mRNA from recycled 40S ribosomal subunits, and was also able to interfere with the formation of the 48S initiation complex promoted by eIF2^5^. A complex between Met-tRNA_i_^Met^ and eIF2D is formed in a GTP-independent fashion. This complex can interact with the 40S ribosomal subunit to deliver the initiator to the P site^29^. Accordingly, eIF2D has been considered as a true initiation factor, though the exact function of this protein in mammalian cells remains enigmatic. Akin to eIF2A, the orthologue of eIF2D is dispensable in yeast^20^,^29^, but comparable studies have not been performed in mammalian cells. Our present results show that human cells with a knock-out for eIF2A or eIF2D are viable and synthesize proteins in a manner similar to wild-type cells. In addition, by investigating the potential involvement of these two proteins for the translation of SINV sgmRNA, we demonstrate that these factors are not required for sgmRNA translation, even when eIF2α is phosphorylated. These findings support the novel proposal that eIF2 is not replaced by a cellular protein during the translation of SINV sgmRNA, instead this viral mRNA has evolved a specialized structure that makes it independent for eIF2. The consequences for the virus life cycle are that significant amounts of structural proteins can be produced upon the translation of sgmRNA even under stress conditions that appear after viral infection.

## Results

### Characterization of cell lines and translation of cellular mRNAs

**Figure 1:**
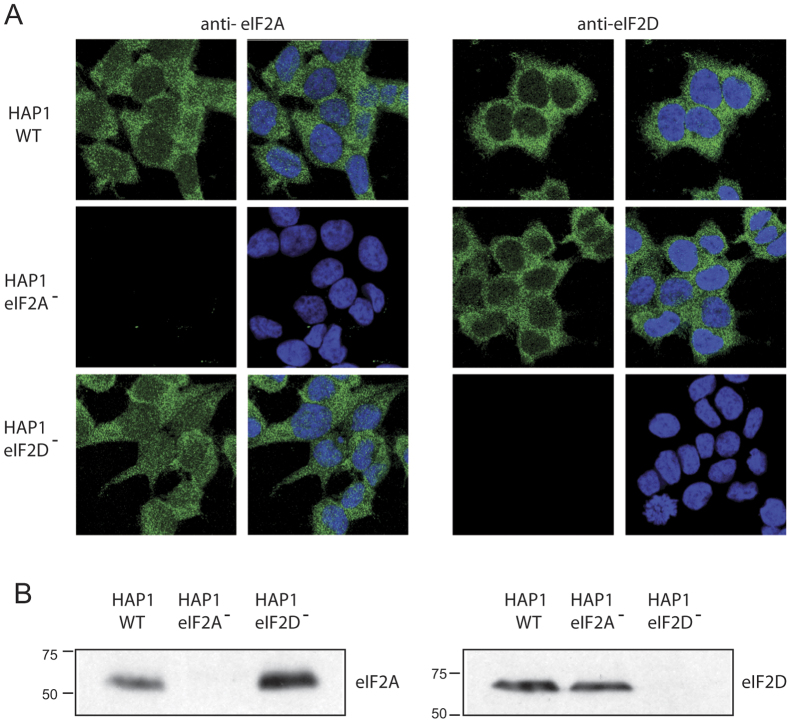
Characterization of the different HAP1 cell lines by immunocytochemistry and western blotting. (**A**) HAP1 WT, HAP1-eIF2A^−^ and HAP1-eIF2D^−^ cells were seeded on coverslips in wells of an L-4 plate, fixed and stained with anti-eIF2A or anti-eIF2D rabbit polyclonal antibodies. The presence and localization of eIF2A and eIF2D (green) were observed by confocal microscopy using secondary anti-rabbit antibodies conjugated to Alexa 488. Nuclei (blue) were stained with Topro-3. (**B**) The presence of eIF2A or eIF2D in HAP1 WT, HAP1-eIF2A^−^ or HAP1-eIF2D^−^ cells was also determined by western blotting with anti-eIF2A and anti-eIF2D antibodies.

We first assessed the viability and cellular translation of WT and KO cell lines. No differences were found in these parameters between HAP1 parental (HAP1 WT) and the two KO cell lines, HAP1-eIF2A^−^ and HAP1-eIF2D^−^, and all cell lines display the same fibroblast-like morphology and grew equally well (results not shown). We next examined the expression of eIF2A and eIF2D by immunocytochemistry using specific antibodies. Double staining of HAP1 WT cells with Topro-3 revealed that eIF2A was clearly expressed in the cytoplasm and a proportion was also found in the nucleus, whereas eIF2D was mainly expressed in the cytoplasm (Fig. 1A). As anticipated, eIF2A was detected both in WT and HAP1-eIF2D^−^ cells, but not in HAP1-eIF2A^−^ cells (Fig. 1A). A similar result was found for eIF2D expression (Fig. 1A). Loss of eIF expression in the respective KO cell lines was verified by western blotting (Fig. 1B). This is the first time that KO cell lines for eIF2A or eIF2D have been obtained in mammalian cells.

**Figure 2:**
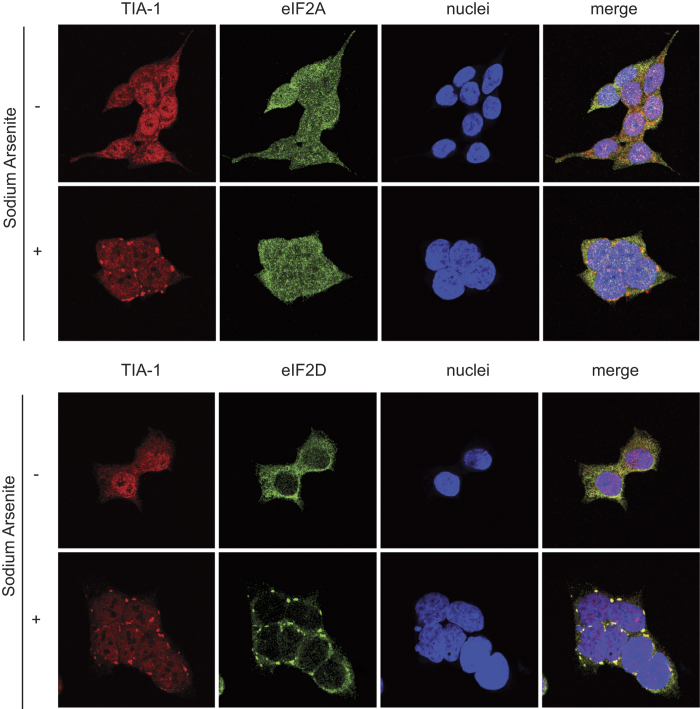
Induction of stress granules by sodium arsenite treatment in HAP1 cell lines. HAP1 WT, HAP1-eIF2A^−^ and HAP1-eIF2D^−^ cells previously seeded on coverslips in wells of an L-4 plate were treated or not with 200 μM arsenite for 1 h and then fixed and permeabilized. Immunodetection was carried out using primary goat anti-TIA-1, rabbit anti-eIF2A or rabbit anti-eIF2D antibodies. An anti-goat antibody conjugated to Alexa 555 was used to detect TIA-1 (red) and anti-rabbit antibodies conjugated to Alexa 448 were employed to detect eIF2A (green) or eIF2D (green). DAPI (4′-6-diamidino-2-phenylindole) was used to stain the nuclei (blue).

We next examined the cellular response to sodium arsenite treatment, which induces endoplasmic reticulum stress response and phosphorylation of eIF2α, resulting in global translation inhibition^30^. As shown in Fig. 2, arsenite treatment of HAP1 WT cells induced the formation of stress granules containing TIA-1 protein, which redistributed from the nucleus to the cytoplasm. Analysis of eIF2A and eIF2D expression in WT cells following arsenite exposure revealed that eIF2D but not eIF2A clearly co-localized with TIA-1 in cytoplasmic stress granules (Fig. 2). Subsequently, the action of arsenite on cellular translation in WT and KO cell lines was measured by radioactive labeling of cellular proteins followed by their detection using SDS-PAGE and fluorography. No differences were found in protein synthesis between the three cell lines (Supplementary Figure 1). Accordingly, arsenite induced a similar concentration-dependent inhibition of cellular translation in all three cell lines, which was particularly profound at 50–100 μM and almost complete at 200 μM (Supplementary Figure 1A). This finding suggests that eIF2A or eIF2D do not substitute, even partially, the action of eIF2 during the initiation of global mRNA translation. Furthermore, the total amount of eIF2α and the degree of eIF2α phosphorylation was comparable between the three cell lines and increased in parallel with the blockade of cellular protein synthesis (Supplementary Figure 1B). In summary, HAP1-eIF2A^−^ and HAP1-eIF2D^−^ cell lines are viable and protein synthesis is blocked by eIF2α phosphorylation to an extent similar to that found in HAP1 WT cells.

### Viral protein synthesis in HAP1 eIF2A and eIF2D KO cells

**Figure 3:**
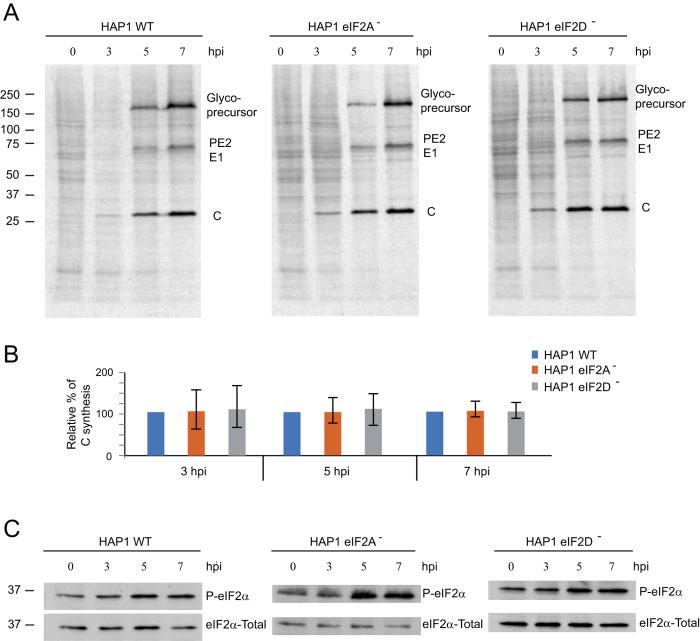
Effect of SINV infection on protein synthesis and eIF2α phosphorylation in HAP1 cell lines. Equal numbers of HAP1 WT, HAP1-eIF2A^−^ and HAP1-eIF2D^−^ cells were infected or not with 10 pfu/cell SINV and labeled with ^35^S-Met/Cys from 3 to 4, 5 to 6 or 7 to 8 hpi. (**A**) After labeling, cells were collected in loading buffer and analyzed by SDS-PAGE and autoradiography to detect protein synthesis. (**B**) Densitometric analysis of C production in WT and KO cells. The graph shows the percentage values in relation to the amount of C synthesized in HAP1 WT cells at different hpi. The results are displayed as mean ± SD of three representative experiments. (**C**) The amount of phospho-eIF2α and total eIF2α was analyzed in parallel by western blotting.

Previous studies testing the involvement of eIF2A on viral mRNA translation have used RNA interference approaches (siRNA) to reduce the amount of eIF2A in cultured cells^17^,^31^. Although instructive, this approach has two major weaknesses: often, residual amounts of eIF2A could partially maintain viral translation and siRNA treatment may have some side-effects on viral replication and/or translation steps. The use of KO cells overcomes these problems, constituting a more robust approach to test the functionality of eIF2A or eIF2D in SINV-infected cells. Thus, WT and KO cell lines were infected with SINV and protein synthesis was analyzed by radioactive labeling at different periods post infection. No differences were found between the three cell lines in the amount or in the kinetics of viral proteins synthesized (Fig. 3A and B), supporting the view that neither eIF2A nor eIF2D are required for SINV sgmRNA translation. Additionally, the absence of these factors did not affect earlier steps of viral replication that are necessary for sgmRNA translation.

**Figure 4:**
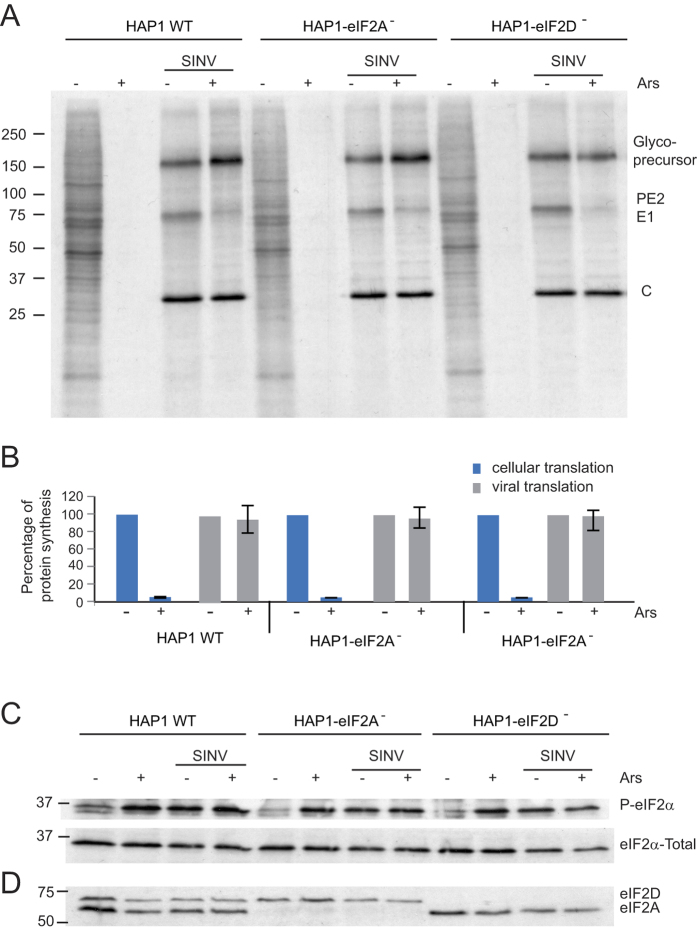
Effect of SINV infection and sodium arsenite treatment on protein synthesis and eIF2α phosphorylation in HAP1 cell lines. Mock-infected or SINV- infected cells from each HAP1 line were treated or not with 200 μM arsenite at 7 hpi during 1 h and 15 min and labeled with ^35^S-Met/Cys during the last hour of treatment. (**A**) Cells were collected in loading buffer and analyzed by SDS-PAGE and autoradiography to detect the protein synthesis. (**B**) Densitometric analysis of cellular and viral proteins synthesized in the absence or presence of arsenite in the different cell lines. The graph shows the percentage values obtained from untreated versus their counterpart cells treated with arsenite. The results are displayed as mean ± SD of three representative experiments. (**C**) The amount of phospho-eIF2α and total eIF2α was analyzed in parallel by western blotting. (**D**) As a control for the presence of eIF2A and eIF2D, the amount of these proteins was also analyzed by western blotting using a mixture of rabbit polyclonal anti-eIF2A and rabbit polyclonal anti-eIF2D as primary antibodies.

It is well established that SINV replication stimulates PKR, leading to eIF2α phosphorylation^7^,^16^,^17^. We therefore analyzed the induction of eIF2α phosphorylation in the three cell lines during SINV infection. The kinetics and the degree of eIF2α phosphorylation were similar in the three cell lines upon infection with SINV (Fig. 3C). Phosphorylation of eIF2α increased at 3 hours post infection (hpi) and reached a maximum after 5–7 h. To further question whether eIF2A or eIF2D could replace eIF2 for the translation of SINV sgmRNA, cells were treated with arsenite, which induces almost 100% phosphorylation of eIF2 in SINV-infected cells (7). At 7 hpi, mock-infected or SINV-infected HAP1 cells were treated with 200 μM arsenite and 15 min later protein synthesis was estimated by radioactive labeling over the next hour. Cellular translation was blocked by arsenite treatment, whereas almost no inhibition was detected in SINV-infected cells (Fig. 4A and B). Viral protein synthesis occurred at similar levels in all three cell lines and was equally resistant to arsenite. Although viral translation was not diminished by arsenite, polyprotein processing was affected leading to an accumulation of the glycoprotein precursor, which is in agreement with previous observations^7^. Consistent with our earlier results (Fig. 3), eIF2α was clearly phosphorylated in all three cell lines infected with SINV (Fig. 4C). eIF2α phosphorylation was maintained upon addition of arsenite, suggesting that virtually all eIF2α was phosphorylated under these conditions. As expected, eIF2A and eIF2D expression was absent in the respective HAP1 KO cell lines (Fig. 4D). Collectively, these results show that neither eIF2A nor eIF2D are necessary for SINV sgmRNA translation, even when eIF2α is phosphorylated.

### Translation of SINV sgmRNA in cells devoid of eIF2A and eIF2D

**Figure 5:**
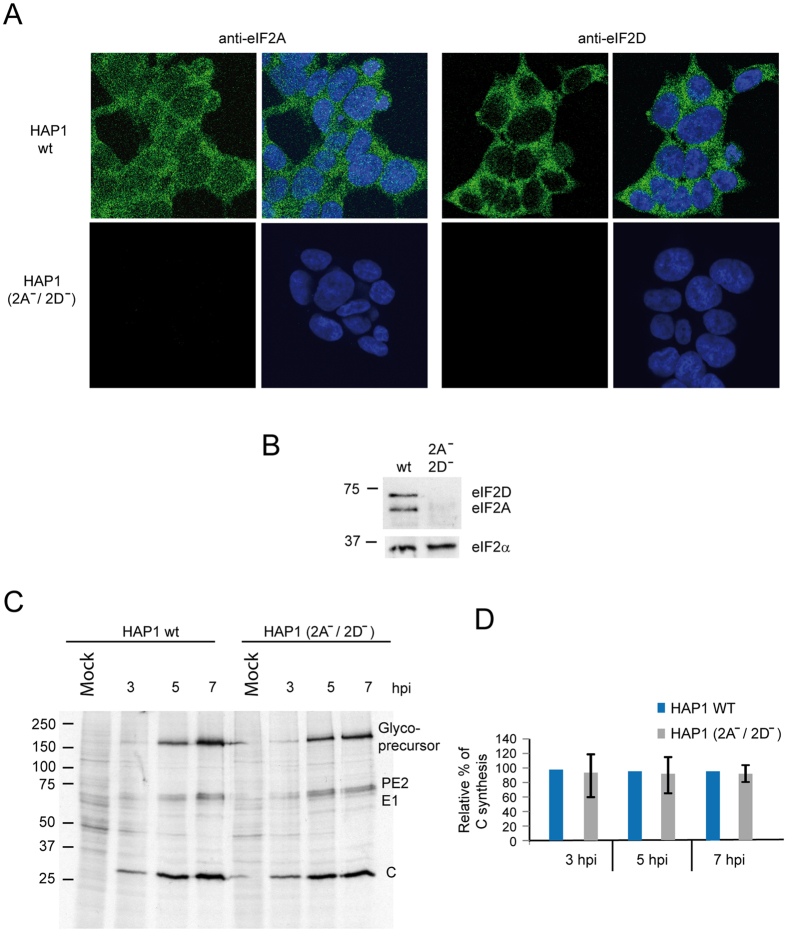
SINV protein synthesis of the double KO cell line HAP1 eIF2A^−^/2D^−^. (**A**) HAP1 WT, and HAP1 (eIF2A^−^/eIF2D^−^) cells were seeded on coverslips in wells of an L-4 plate, fixed and stained with anti-eIF2A or anti-eIF2D rabbit polyclonal antibodies. The presence and localization of eIF2A and eIF2D (green) were observed by confocal microscopy using secondary anti-rabbit antibodies conjugated to Alexa 488. Nuclei (blue) were stained with Topro-3. (**B**) Western blotting analysis using a mixture of rabbit polyclonal anti-eIF2A and rabbit polyclonal anti-eIF2D as primary antibodies and anti-rabbit immunoglobulin G antibody coupled to peroxidase as secondary antibodies in lysates of HAP1 WT and HAP1 (eIF2A^−^/eIF2D^−^) cells. (**C**) Equal numbers of HAP1 WT and HAP1 (eIF2A^−^/eIF2D^−^) cells were infected or not with 10 pfu/cell of SINV and labeled with ^35^S-Met/Cys from 3 to 4, 5 to 6 or 7 to 8 hpi. Then, cells were collected in loading buffer and analyzed by SDS-PAGE and autoradiography to detect protein synthesis. (**D**) Densitometric analysis of C production in WT and double KO cells. The graph shows the percentage values in relation to the amount of C synthesized in HAP1 WT cells at different hpi. The results are displayed as mean ± SD of three representative experiments.

**Figure 6:**
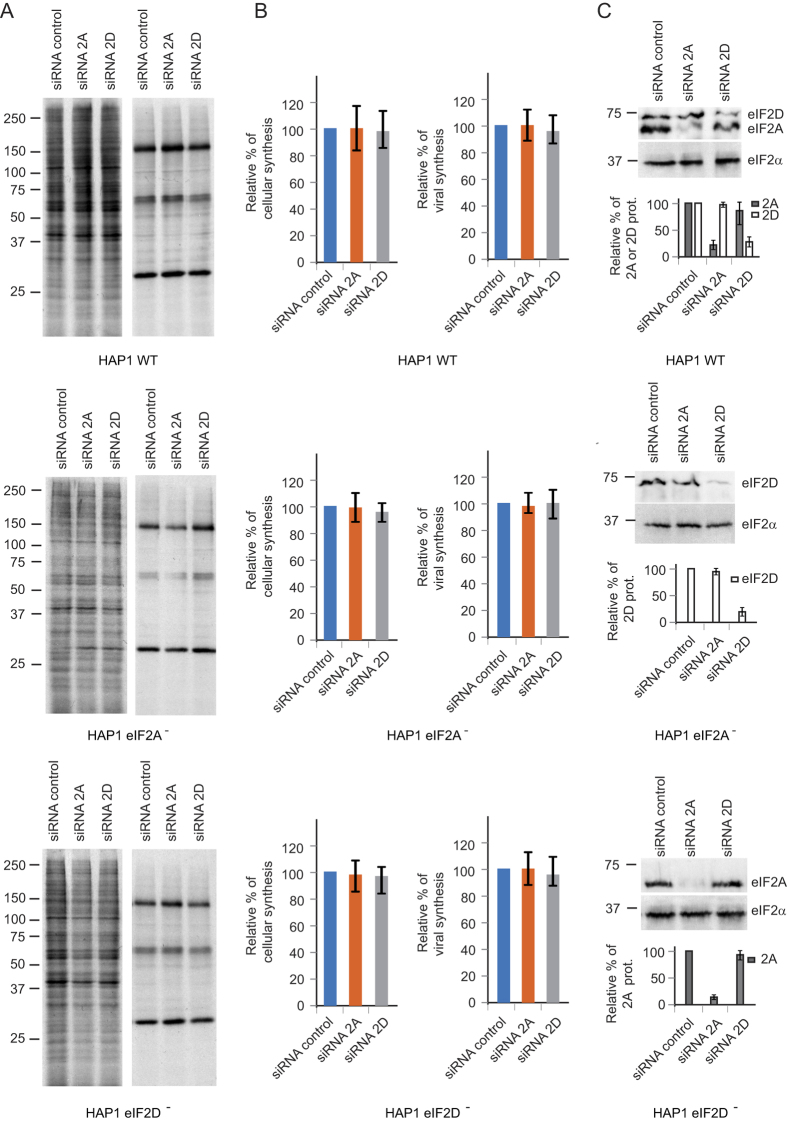
Effect of silencing eIF2A or eIF2D on SINV sgmRNA translation. HAP1 WT, HAP1-eIF2A^−^ and HAP1-eIF2D^−^ cells were treated with a mixture of siRNAs against eIF2A, eIF2D or control siRNAs and at 42 hpt cells were infected or not with SINV (10 pfu/cell). (**A**) Protein synthesis was analyzed from 6 to 7 hpi by radioactive labeling and SDS-PAGE. (**B**) Densitometric analysis of cellular and viral proteins synthesized in the siRNA-treated cells. The graphs show the percentage values obtained from cells treated with siRNAs against eIF2A or eIF2D versus their counterpart cells treated with control siRNAs. The results are displayed as mean ± SD of three representative experiments. (**C**) The degree of depletion of eIF2A or eIF2D was analyzed in parallel by western blotting using a mixture of rabbit polyclonal anti-eIF2A and rabbit polyclonal anti-eIF2D as primary antibodies. As a loading control, the amount of eIF2α was also determined (upper panel). Densitometric analysis of the amount of eIF2A or eIF2D in the siRNA-treated cells. The graph shows the percentage values obtained from cells treated with siRNAs against eIF2A or eIF2D versus their counterpart cells treated with control siRNAs. The results are displayed as mean ± SD of three representative experiments (lower panel).

The possibility that eIF2A could be replaced by eIF2D and vice versa, although unlikely, in the single KO cell lines studied above was next evaluated. To do this, we used a double KO HAP1 cell line deficient for both eIF2A and eIF2D. Viability and morphology of this cell line was similar to that of wild-type HAP1 cells. As expected, HAP1 eIF2A^−^/2D^−^ cells did not express eIF2A or eIF2D as revealed by immunocytochemistry and by western blotting using specific antibodies against these proteins (Fig. 5A and B). Next, translation of sgmRNA in these cells was assayed at different times after SINV infection by radioactive labeling and SDS PAGE. As shown in Fig. 5C and D, SINV infection of HAP1 eIF2A^−^/2D^−^ resulted in a rapid inhibition of cellular translation and the synthesis of late viral proteins directed by sgmRNA to levels comparable to those observed with HAP1 WT cells. These findings are conclusive and are consistent with the notion that neither eIF2A nor eIF2D participate in the initiation of sgmRNA translation. As a complementary test to determine whether eIF2A or eIF2D participate in SINV sgmRNA translation, we used a gene silencing approach to knock-down these proteins. As stated earlier, a potential pitfall of this approach is that residual amounts of initiation factor remain after silencing. Nevertheless, it serves to bolster the experiments using KO cell lines since siRNAs block translation of the corresponding mRNA and, in principle, no truncated initiation factors are synthesized. Cell lines were mock- or SINV-infected 42 h after transfection of the corresponding HAP1 cell lines with siRNAs. Protein synthesis was measured by radioactive labeling at 6–7 hpi and analyzed by SDS PAGE (Fig. 6A and B). Once again, translation of SINV sgmRNA was clearly apparent under all the conditions tested. siRNAs depleting eIF2A or eIF2D in HAP1 WT cells failed to block SINV protein synthesis. Furthermore, HAP1 eIF2A^−^ cells transfected with siRNA for eIF2D also synthesized viral proteins at control levels. A similar situation was found when HAP1 eIF2D^−^ cells were transfected with siRNA to deplete eIF2A. Therefore, the depletion of eIF2A or eIF2D in HAP1 WT or in the KO cell lines has no detrimental effects on sgmRNA translation. The amount of eIF2A or eIF2D present 48 h after siRNA transfection in the three cell lines was analyzed by western blotting. Densitometric analysis indicated that eIF2A was silenced by 83% in HAP1 WT and 99% in HAP1 eIF2D^−^, whereas eIF2D was silenced by 81% in HAP1 WT and 85% in HAP1 eIF2A^−^ (Fig. 6C). These results clearly indicate that depletion of eIF2A or eIF2D does not abrogate the synthesis of viral proteins directed by sgmRNA and are consistent with the findings described using KO cell lines.

### Protein synthesis directed by SINV sgmRNA lacking the initiator AUG codón

**Figure 7:**
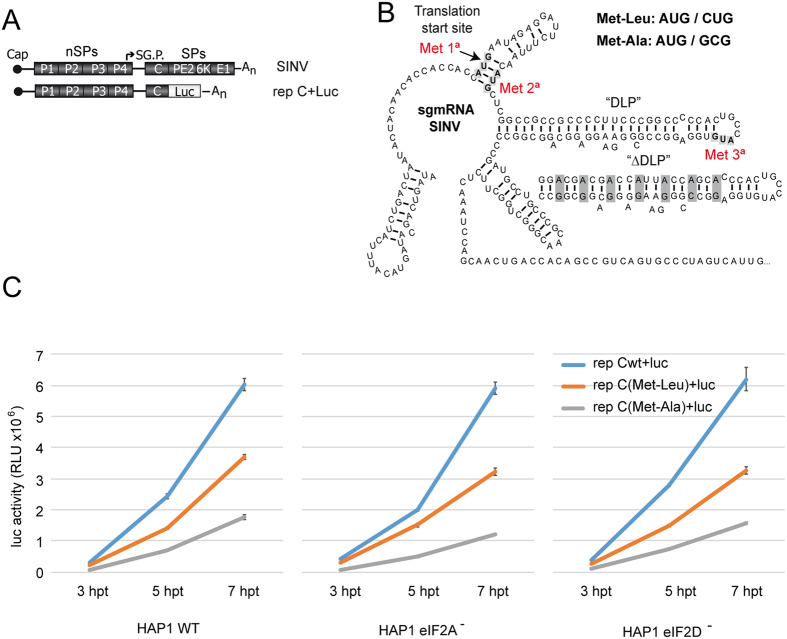
Translation of sgmRNA WT or mutated at the initiation codon in HAP1 cell lines transfected with SINV replicons. (**A**) Schematic representation of the SINV genome and the replicon wt used to make the different variants tested. (**B**) Schematic representation of the secondary structure of SINV sgmRNA. The AUGs mutated in the variants Met-Leu or Met-Ala are highlighted as well as the third in-frame AUG (the first and the second AUGs were mutated to CUG or GCG, respectively). Modified DLP structure, ΔDLP is also illustrated. (**C**) HAP1 WT, HAP1-eIF2A^−^ and HAP1-eIF2D^−^ cells were transfected with *in vitro* synthesized replicons rep C + luc, rep C + luc (Met-Leu) or rep C + luc (Met-Ala), and cells were recovered to measure luciferase activity at different periods post transfection. Luciferase activity results are displayed as mean ± SD of three representative experiments performed in triplicate.

**Figure 8:**
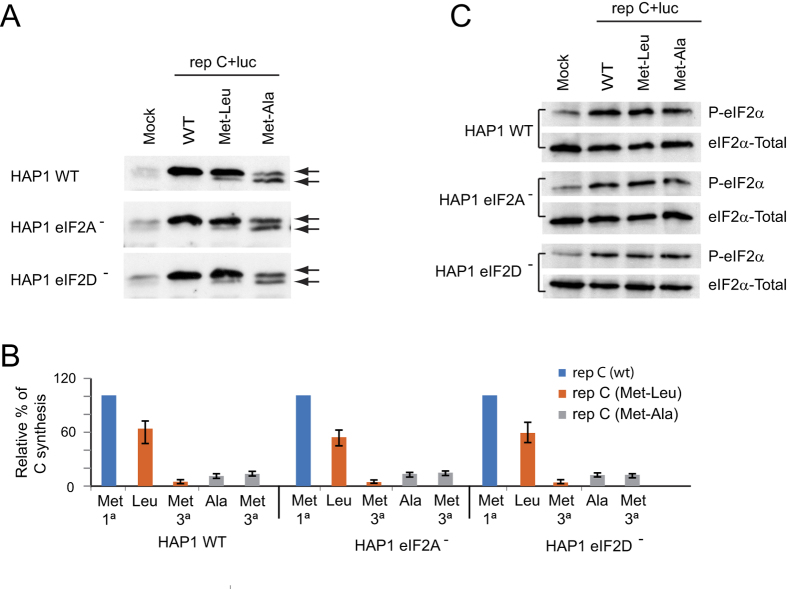
Western blot analysis of SINV protein C and phosphorylation of eIF2α. (**A**) HAP1 WT, HAP1-eIF2A^−^ and HAP1-eIF2D^−^ cells were transfected with *in vitro* synthesized replicons rep C + luc, rep C + luc (Met-Leu) or rep C + luc (Met-Ala). At 7 hpt, cells were collected in loading buffer and analyzed by western blotting with an anti-C antibody. The mobilities of the C products derived from the different replicons are indicated by arrows. (**B**) Densitometric analysis of the different C proteins synthesized from rep C + luc (Met-Leu) or rep C + luc (Met-Ala). The graph shows the percentage values in relation to the amount of C synthesized by rep C + luc in each cell line. The results are displayed as mean ± SD of three representative experiments. (**C**) Analysis of eIF2α phosphorylation and total eIF2α by western blotting using specific antibodies as described in Materials and Methods.

In eukaryotes, a number of proteins are synthesized starting at non-AUG codons^32^,^33^. Recent evidence has implicated eIF2A in the initiation of translation on non-AUG codons in mammalian cells^23^,^24^. Because SINV sgmRNA can still direct translation even when the initiator AUG codon has been changed to other codons^7^, we questioned whether this initiation was mediated by a mechanism involving eIF2A or eIF2D. To do this, we examined SINV replicons bearing sgmRNAs with altered AUG_i_ codons (see scheme in Fig. 7A and B). Thus, AUG_i_ was modified to CUG (encoding Leu) or GCG (encoding Ala), rendering rep C + luc (Met-Leu) or rep C + luc (Met-Ala), respectively. These replicons were obtained by *in vitro* transcription of the corresponding plasmids and were transfected into the three HAP1 cell lines. We initially tested the kinetics of luciferase production and found that its synthesis from each replicon was very similar in the three cell lines; however, luciferase production was 50–60% (Met-Leu) and 20–25% (Met-Ala) relative to control (wt) values (Fig. 7C). Remarkably, this inhibition was similar in the three cell lines. Thus, the absence of eIF2A or eIF2D did not affect sgmRNA translation with the replicons when the AUG_i_ was changed to CUG or GCG. Previously, we found that the initiation of translation in SINV sgmRNA variants that do not contain AUG_i_ takes place at the mutated AUG_i_ and at the following AUG that appears in the C coding sequence^7^. To assess the start site of C synthesis, the different forms of this protein were analyzed by western blotting. The second AUG of the open reading frame of C from these variants was also mutated to distinguish easily by electrophoretic separation the products derived from initiation at the alternative codons because the protein derived from the translation at the AUG (3° in the wt sequence) yields a product with 20 amino acids less as compared to genuine C. As shown in Fig. 8A and B, rep C + luc synthesized genuine C protein, whereas rep C + luc (Met-Ala) synthesized similar amounts of two C products of different mobility. These two different forms of C differ in about 20 aminoacids, according to the expected products derived from the initiation at the alternative codon GCG (first AUG mutation) or third AUG. Moreover, rep C + luc (Met-Leu) rendered two C products, but the amount of genuine C, which presumably initiates at CUG, was more than 90% of the total. Again, no differences were observed regarding the different C products synthesized in WT and the different KO cell lines tested. These results indicate that neither eIF2A nor eIF2D are involved in the initiation of sgmRNA translation when CUG or GCG replaces AUG_i_. Previous observations from our laboratory have demonstrated that SINV replicons induce the phosphorylation of eIF2α in a way akin to SINV infection [15]. We therefore used western blotting to assess whether the replicons described above also induce eIF2α phosphorylation in the HAP1 cell lines. Indeed, transfection of the SINV replicons induced the phosphorylation of eIF2α in a similar manner in the three cell lines (Fig. 8C).

**Figure 9:**
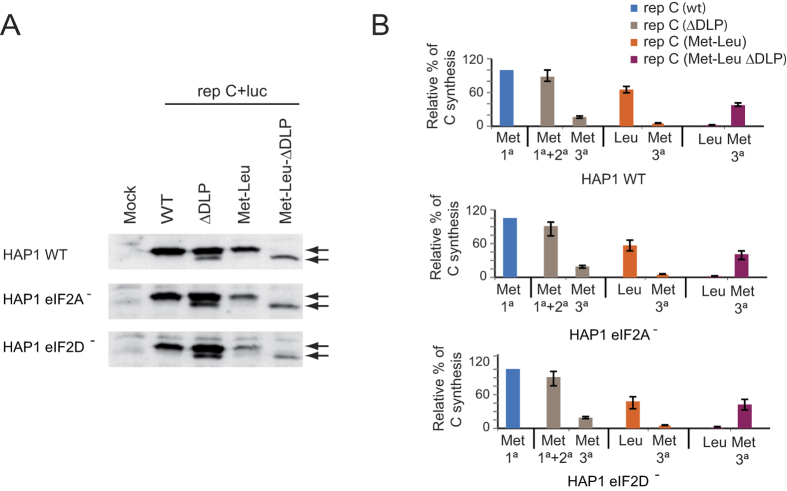
Involvement of the DLP hairpin in signaling the initiation codon. (**A**) HAP1 WT, HAP1-eIF2A^−^ and HAP1-eIF2D^−^ cells were transfected with *in vitro* synthesized replicons rep C + luc, rep C + luc (ΔDLP), rep C + luc (Met-Leu) or rep C + luc (Met-Leu-ΔDLP). The DLP structure and the mutations introduced to generate the variant ΔDLP are indicated in Fig. 7B. At 7 hpt, cells were collected in loading buffer and analyzed by western blotting with an anti-C antibody. The mobilities of the C products derived from the different replicons are indicated by arrows. (**B**) Densitometric analysis of the different C proteins synthesized by the replicons in relation to the canonical product of C synthesized by rep C + luc in each cell line. The results are displayed as mean ± SD of three representative experiments.

The finding that rep C + luc (Met-Leu) used CUG in place of AUG_i_ quite efficiently while the third AUG of sgmRNA was practically ignored was striking. Since one of the functions of the DLP is to signal the precise codon to start translation, we decided to analyze the functioning of the DLP in the sgmRNA variant encoding Leu in place of Met. We generated a new construct bearing CUG as the initiation codon, followed by an unstructured DLP: rep C + luc (Met-Leu-ΔDLP) (see Fig. 7A and B). The corresponding replicative RNA was transfected into HAP1 cells and C protein production was analyzed by western blotting. As controls, we used rep C + luc, which renders a genuine C protein, and an unstructured DLP in rep C + luc (ΔDLP), which leads to a loss of fidelity in the election of the AUG_i_. Thus, several C products are produced that initiate at different codons as a result of leaky scanning^7^,^17^. We found that rep C + luc (Met-Leu) almost entirely initiated at CUG, giving rise to a C protein of the same mobility as the control (Fig. 9). Notably, the presence of the unstructured DLP in the rep C + luc (Met-Leu-ΔDLP) construct abrogated the initiation at CUG and almost all of the C generated initiated at the third AUG codon (Fig. 9). Nonetheless, the recognition of CUG and the initiation at the third AUG codon when DLP was unstructured, were similar in all three HAP1 cell lines tested, further demonstrating that neither eIF2A nor eIF2D are involved in the recognition of the initiation codon in sgmRNA and in the functioning of the DLP.

## Discussion

Several mRNAs from animal viruses are able to direct translation even after phosphorylation of eIF2α^34^,^35^,^36^. In most cases, however, the precise mechanism by which the initiation event occurs when eIF2 is inactivated is unclear. An extreme case of initiation of mRNA translation in the absence of eIFs, including eIF2, is represented by mRNAs bearing an IRES in the intergenic region in Cricket paralysis virus (CrPV)^37^. In this setting, the IRES is a folded structure that mimics tRNA and can interact with the decoding A site of the ribosome^38^,^39^. The IRES is translocated to the P site by eEF2, leaving the A site free and the first codon ready to start translation. Other animal viruses including picornaviruses that contain an IRES element can initiate translation by a dual mechanism. Early during infection, intact eIF2 is necessary to initiate viral protein synthesis, whereas at late periods mRNA translation occurs even when eIF2 is phosphorylated^40^,^41^,^42^. In this case, picornavirus proteases confer eIF2 independence for IRES-driven translation^41^,^43^,^44^. However, the precise mechanism by which picornaviruses initiate translation without eIF2 remains to be elucidated. Hepatitis C virus provides another example of eIF2-independent translation driven by an IRES element^35^,^45^. In this context, several factors have been suggested to replace eIF2, including eIF5B, eIF2D or eIF2A^5^,^29^,^31^,^46^. A different example of eIF2-independent translation is provided by the capped sgmRNA from alphaviruses^7^,^16^,^17^. In this case, the hairpin located 24 nt downstream of the AUG_i_ is required to initiate translation without active eIF2. The precise functioning of this hairpin during initiation remains enigmatic. Initially, a stable hairpin structure was noticed in the coding region of sgmRNA, that enhaced its translation^15^. It was speculated that this hairpin stalled ribosomes leaving the AUG_i_ at the P site. However, this possibility seems unlikely because for this to occur the hairpin should be located at 14 nt downstream the initiation codon^47^. It was later observed that when eIF2α does not become phosphorylated, translation of the sgmRNA takes place without the integrity of this hairpin^16^,^17^. Another model for the function of DLP is that it interacts with the ribosomal P site in a manner similar to that described for CrPV^6^. The possibility that eIF2 is replaced by eIF2A has also been proposed based on gene silencing^17^. However, this possibility seems unlikely in view of our present findings since we demonstrate that sgmRNA is efficiently translated in HAP1 cells lacking eIF2A, even when eIF2α is phosphorylated. Moreover, our present observations on the effect of silencing eIF2A on sgmRNA translation clearly indicate that this factor is not necessary in the human cell line analyzed. Also, the suggestion that eIF2D (previously known as ligatin) participates in protein synthesis directed by SINV sgmRNA is not supported by our present results. It seems clear from our findings that cells lacking eIF2D and active eIF2 are infected with SINV and synthesize viral late proteins at levels similar to those of controls. Therefore, whereas the precise mechanism of protein synthesis directed by sgmRNA in the absence of active eIF2 remains to be resolved, we are confident that eIF2A or eIF2D are dispensable for this process.

An additional function of the hairpin DLP is to signal the start codon of sgmRNA in such a manner that the change of AUG_i_ to other codons engenders sgmRNA functional, albeit to a lower extent^7^. We show here that the substitution of CUG (leucine) for AUG_i_ has a moderate effect on viral protein synthesis directed by this mRNA variant. Indeed, only 40–50% inhibition of C synthesis was observed. Since binding of the ternary complex GTP-eIF2-Met-tRNA_i_^Met^ is only promoted by AUG codons, the synthesis of C protein initiating at Leu will take place without eIF2. Interestingly, even in this case neither eIF2A nor eIF2D were required for this non-AUG initiation of translation since the level of the production of C protein was similar in the three cell lines transfected with the rep C + luc (Met-Leu). However, the disorganization of the DLP has profound effects with regards to the initiation codon used. In rep C + luc (ΔDLP), initiation was observed at the first AUG_i_ and also at the second and third AUGs, indicating that the genuine DLP structure is important to signal the correct initiation codon. Notably, initiation at CUG in cells transfected with rep C + luc (Met-Leu-ΔDLP) was abrogated when the DLP was unstructured. In this case, a truncated C protein was synthesized that mainly starts at the third AUG, and a similar pattern was found in the three cell lines assayed. Therefore, we can conclude that the structure of the DLP hairpin is more important for initiation codon selection than the presence of eIF2A or eIF2D. In sharp contrast to previous reports, the possibility that now arises is that eIF2 is not substituted by any other factor to translate sgmRNA in SINV infected cells. It could even be possible that the DLP itself could carry out this function. We have previously proposed that the DLP directly interacts with the 40S ribosomal subunit or the 80S ribosome at either the A- or P-site, resembling in this regard the initiation event followed by the IGR IRES of CrPV^6^,^37^,^38^. The suggestion that Semliki Forest virus DLP can interact with a sequence present in 18S rRNA to signal the initiation start codon is interesting^48^; however, the speculation that eIF4A participates in the unwinding of DLP after this interaction is not supported by the evidence that selective inhibitors of eIF4A, such as hippuristanol or pateamine A, do not influence initiation of SINV sgmRNA^18^,^49^. Our current observations support the concept that eIF2 is not replaced by cellular proteins, instead the acquisition of the DLP structure during alphavirus evolution led to eIF2 independent translation. It is known that the presence of DLP structure allows the translation of sgmRNA when eIF2 is phosphorylated^16^,^17^,^18^. This is important for the virus biology because large amounts of structural proteins have to be synthesized under stress conditions. It is well established that alphavirus infection induces the phosphorylation of eIF2α upon infection of mammalian cells. Therefore, the virus has evolved the DLP within the coding region of sgmRNA to be translated under stress conditions that appear after infection. Curiously, the DLP structure also allows the initiation of the translation using alternative codons as GCG or CUG. This represents a unique example of a viral mRNA that is capped, is translated following the scanning mechanism and still does not utilize eIF2 during the initiation process^6^. Thus, the functional replacement of eIF2 and the ternary complex by a viral RNA structure may be a common mechanism employed by a variety of animal viruses including those that contain an IRES in their mRNAs. Further experiments aimed to elucidate this step during the initiation of SINV sgmRNA are needed. Nonetheless, the important conclusion of our present observations is that neither eIF2A nor eIF2D substitute for eIF2 and are not required to initiate translation of this viral mRNA.

## Methods

### Cell lines and viruses

Wild-type (WT) HAP1 human haploid cells and HAP1 cells knocked-out for eIF2A (cat# HZGHC002650c001), eIF2D (cat# HZGHC002652c005) or double knock-out for eIF2A and eIF2D (HZGHC005122c010) were purchased from Horizon Discovery Group plc. The eIF2A knock-out (KO) cell line (gi|977380191|ref|NM_032025.4|) has a 16 bp deletion in exon 4 resulting in a frameshift that generates a protein of 108 aa rather than 585 aa of the WT protein. The eIF2D KO cell line (gi|56699484|ref|NM_006893.2|) has a 10 bp deletion in exon 3 resulting in a frameshift that generates a protein of 103 aa rather than 584 aa of the WT protein. The double KO line has the same 16 bp deletion in exon 4 of the single eIF2A KO cell line and a 22 bp deletion in exon 3 of eIF2D that generates a protein of 99 aa rather than 584 aa of the WT protein. Cells were cultured in IMDM (Invitrogen) supplemented with 10% fetal calf serum. SINV stock was obtained from a pT7 SV WT infective cDNA clone^50^. Titers of viruses were determined by plaque assay.

### Plasmids and constructs

SINV replicons expressing C and luciferase were obtained by *in vitro* transcription from plasmids derived from pT7 SV wt^50^. pT7 rep C + luc^51^ and pT7 rep C + luc ΔDLP^18^ have been described previously. pT7 rep C + luc (Met-Ala), pT7 rep C + luc (Met-Leu) and pT7 rep C + luc (Met-Leu-ΔDLP) were constructed for the present study. To generate pT7 rep C + luc (Met-Ala), the Hpa I/Aat II digestion fragment from pT7 rep C (Met-Ala)^7^ was cloned into the corresponding sites of pT7 rep C + luc. pT7 rep C + luc (Met-Leu) was designed as the variant Met-Ala, but in this case both the first and second ATGs of the 26S sequence were mutated to GTGs. pT7 rep C + luc (Met-Leu-ΔDLP) has an altered DLP sequence (ΔDLP) in addition to the GTG mutations.

### *In vitro* RNA transcription and transfection

Plasmids digested with Xho I were used as templates for *in vitro* RNA transcription with T7 RNA polymerase (New England Biolabs) in reactions containing the m^7^G(5′)ppp(5′)G cap analog (New England Biolabs). *In vitro*-synthesized RNAs were treated with DNase I and then transfected in cells using Lipofectamine 2000 reagent (Invitrogen).

### Analysis of protein synthesis by radioactive labeling

Protein synthesis was measured by incubating cells in 0.2 ml DMEM without methionine and cysteine, supplemented with 1 μl EasyTag^TM^ EXPRESS ^35^S protein labeling mix, [^35^S]Met/Cys (11 mCi ml^−1^, 37.0 Tbq mmol^−1^; Perkin Elmer) per well of a 24-well plate for 60 min. Cells were collected in loading buffer (62.5 mM Tris-HCl pH 6.8, 2% SDS, 0.1 M dithiothreitol, 17% glycerol and 0.024% bromophenol blue) and autoradiographic analysis was performed following SDS-polyacrylamide gel electrophoresis.

### Measurement of luciferase activity

Cells were lysed in a buffer containing 0.5% Triton X-100, 25 mM glycylglycine pH 7.8, 1 mM dithiothreitol and complete, EDTA-free, protease inhibitor cocktail (Roche). Luciferase activity was detected using the Luciferase Assay System (Promega) in a Monolight 2010 luminometer (Analytical Luminescence Laboratory).

### Antibodies

Primary antibodies used in this work included a rabbit polyclonal antibody raised against purified SINV C protein generated in our laboratory. Rabbit polyclonal anti-TIA-1 (C-20): sc-1751 and rabbit polyclonal anti-eIF2α antibodies were purchased from Santa Cruz Biotechnology. Rabbit polyclonal anti-eIF2D antibody was purchased from Proteintech, rabbit polyclonal anti-eIF2A antibody was purchased from Bethyl Laboratories Inc., and a rabbit polyclonal antibody raised against phospho-eIF2α (serine 51) was purchased from Cell Signaling Technology.

### Immunocytochemistry and confocal microscopy

Fixation, permeabilization and confocal microscopy were performed as described^52^ using the LSM 710 confocal laser scanning and multiphoton microscope coupled to an inverted microscope (Axio Observer, Zeiss). Primary antibodies were detected by secondary antibodies coupled to Alexa 488 or Alexa 555. Nuclei were stained with Topro-3 or DAPI (4′-6-diamidino-2-phenylindole). All images were collected and analyzed using Zeiss ZEN 2010 software.

### Western blotting

Cells were collected in sample buffer, boiled for 5 min and processed by SDS-PAGE. After electrophoresis, proteins were transferred to a nitrocellulose membrane. Specific rabbit polyclonal antibodies raised against phospho-eIF2α (Ser 51), total eIF2α, SINV Capsid, eIF2A and eIF2D were used at 1:1000 dilution in PBS with 3% BSA and 0.1% Tween 20, except when phospho-eIF2α was analyzed we instead used TTBS (Tris-buffered saline, 0.1% Tween 20). Anti-rabbit immunoglobulin G antibody coupled to peroxidase (Amersham) was used at a 1:5000 dilution. Protein bands were visualized with the ECL detection system (Amersham).

#### siRNA transfection

For transient transfections, siRNAs targeting specifically eIF2A (L-014766-01-0005, Dharmacon), eIF2D (L-003680-01-0005, Dharmacon) or a control siRNA (D-001810-01-05, Dharmacon) were transfected with Lipofectamine 2000 reagent (Invitrogen) according to the manufacturer’s instructions. At 42 hours post-transfection, cells were infected or not with SINV (multiplicity of infection, 10). Protein synthesis was analyzed from 6 to 7 hours post infection by radiactive labeling and SDS-PAGE and the degree of depletion of eIF2A and eIF2D was assessed by western-bloting.

## Additional Information

**How to cite this article:** Sanz, M. A. *et al*. Translation of Sindbis Subgenomic mRNA is Independent of eIF2, eIF2A and eIF2D. *Sci. Rep.* **7**, 43876; doi: 10.1038/srep43876 (2017).

**Publisher's note:** Springer Nature remains neutral with regard to jurisdictional claims in published maps and institutional affiliations.

## Supplementary Material

Supplementary Figure 1
