# Supplementary material for: Translation of Sindbis Subgenomic mRNA is Independent of eIF2, eIF2A and eIF2D
Source: Sci Rep. 2017 Feb 27;7:43876. doi: 10.1038/srep43876 (PMC5327398; doi:10.1038/srep43876)
Supplement: Supplementary Figure 1 [file srep43876-s1.pdf]

Supplementary Info

Translation of Sindbis Subgenomic mRNA is Independent of eIF2, eIF2A and eIF2D.

Miguel Angel Sanz*, Esther González Almela and Luis Carrasco

Centro de Biología Molecular Severo Ochoa (CSIC-UAM). Universidad Autónoma de Madrid.

28049, Madrid. Spain

*Correspondence: +34-91-1964517; masanz@cbm.csic.es

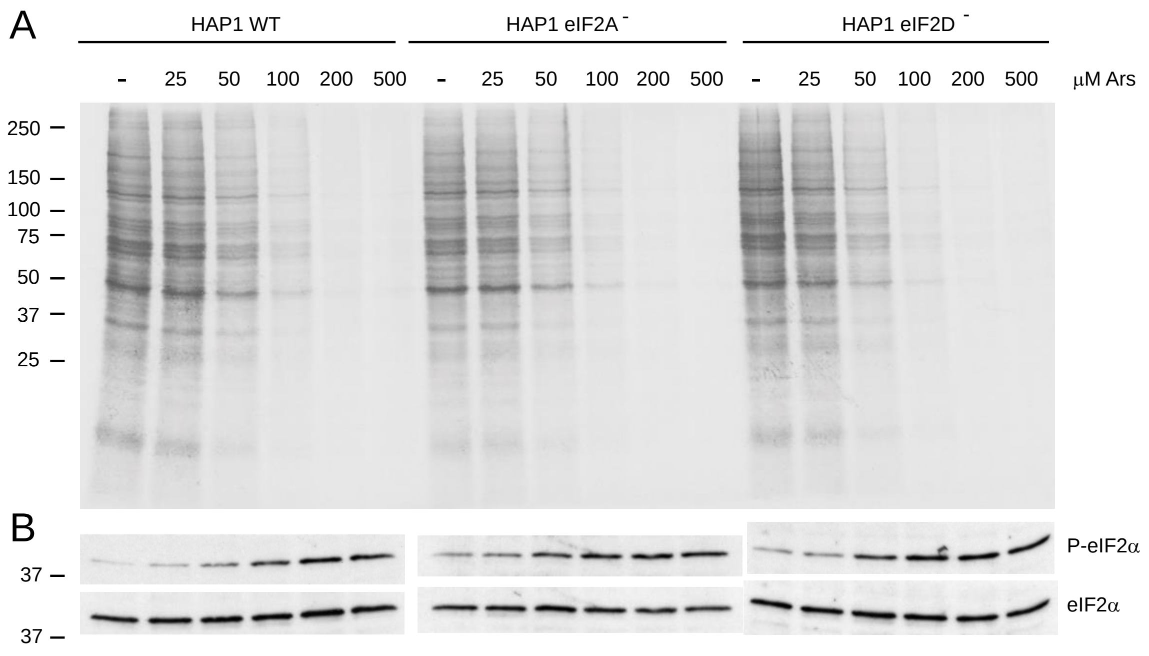

Supplementary Figure 1. Effect of sodium arsenite treatment on protein synthesis and eIF2α phosphorylation in HAP1 cell lines. A) HAP1 WT, HAP1-eIF2A$^-$ and HAP1-eIF2D$^-$ cells previously seeded on coverslips in wells of an L-24 plate were left untreated or treated with different amounts of arsenite for 1 h and 15 min in labeling medium. Protein synthesis was analyzed by radioactive labeling with ^{35}S-Met/Cys during the last hour of treatment followed by SDS-PAGE and autoradiography. **B)** The amount of phospho-eIF2α and total eIF2α was analyzed in parallel by western blotting.
